# NiFe-LDH as a bifunctional electrocatalyst for efficient water and seawater electrolysis: enhanced oxygen evolution and hydrogen evolution reactions†

**DOI:** 10.1039/d5na00350d

**Published:** 2025-07-30

**Authors:** Xin Li, Song-lin Xu, Jia Li, Shuang-shuang Zhang, Bo-yao Zhang, Rong-da Zhao, De-peng Zhao, Fu-fa Wu

**Affiliations:** a School of Materials Science and Engineering, Liaoning University of Technology Jinzhou Liaoning 121000 China Rongdazhaoln@126.com; b School of New Energy, Shenyang Institute of Engineering Shenyang Liaoning 110136 P. R. China hellodepeng@163.com

## Abstract

Water electrolysis for hydrogen production has garnered significant attention due to its high efficiency, environmental friendliness, and abundant resource availability. Developing cost-effective, efficient, and stable materials for water electrolysis is crucial. This study investigates NiFe-LDH, a highly efficient electrocatalyst for the oxygen evolution reaction (OER) in alkaline electrolytes and a bifunctional electrocatalyst for alkaline seawater electrolysis. Its unique layered structure and large specific surface area provide abundant active sites. The prepared NiFe-LDH-4 catalyst exhibits excellent OER performance in 1.0 M KOH electrolyte, requiring only 235 mV overpotential at a current density of 50 mA cm^−2^ and demonstrating a Tafel slope of 80.32 mV dec^−1^. In alkaline seawater electrolyte, it maintains outstanding OER performance while also showing excellent hydrogen evolution reaction (HER) capabilities. Compared to 1.0 M KOH electrolyte, the hydrogen evolution overpotential at −10 mA cm^−2^ current density decreased by 88.5 mV, with the Tafel slope reduced by 53.6 mV dec^−1^. Meanwhile, OER maintained excellent stability in a 1.0 M KOH electrolyte at a current density of 500 mA cm^−2^, and an AEMWE device was constructed. The NiFe-LDH electrocatalyst demonstrates exceptional catalytic activity and high stability, making it a promising candidate for industrial-scale production.

## Abstract 2

In this work, NiFe-LDH nanospheres were prepared by a simple hydrothermal synthesis method. The synthesized product can be directly used as a dual functional electrocatalyst for efficient water and seawater electrolysis.

## Graphical abstract

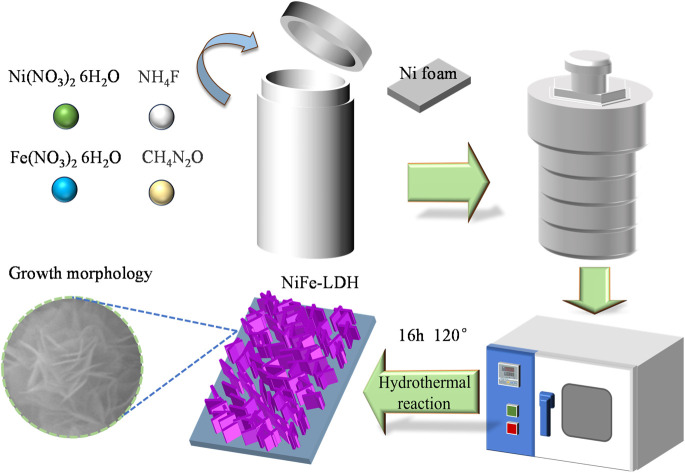

## 1. Introduction

In recent years, as energy and environmental issues have become increasingly severe, renewable and clean energy sources have garnered widespread attention.^1^ Hydrogen, with its high energy density, zero pollution, versatile applications, and easy accessibility, has emerged as a highly promising new energy source.^2–4^ Electrochemical water splitting is a particularly promising technology for hydrogen production. With the assistance of efficient electrocatalysts, this technology can achieve high energy conversion efficiency and mass activity. It demonstrates high efficiency and long-lasting performance at low overpotentials, making it particularly promising in the hydrogen energy field. The two key reactions in electrochemical water splitting are the oxygen evolution reaction (OER) and the hydrogen evolution reaction (HER).^5^ However, the slow kinetics of HER and the large overpotential of OER, along with the complex four-electron oxidation process, severely hinder the practical application of water splitting.^6^ Therefore, there is an urgent need to develop bifunctional electrocatalysts with high catalytic activity and stability for both HER and OER to enhance water splitting efficiency.^7–11^ However, from a practical application perspective, the catalytic activity of a catalyst is not the sole evaluation criterion. Its economic feasibility, particularly the overall consideration of cost and benefit, is equally critical for the large-scale commercialization of the technology.^12–14^ Currently, noble metals (Pt/C, RuO_2_, IrO_2_, *etc.*) are widely recognized as excellent catalysts for OER and HER. However, their low abundance and high cost greatly limit their large-scale application.^15–17^ Consequently, developing low-cost, resource-abundant electrode materials with high catalytic activity and stability is crucial. Non-noble metal sulfides, nitrides, phosphides, hydroxides, and layered double hydroxides (LDHs) have shown significant potential in oxygen and hydrogen evolution fields. Among various candidate materials, nickel-iron layered double hydroxide (NiFe-LDH) is considered one of the most promising alternatives to noble metal catalysts due to its extremely low raw material cost, simple and scalable synthesis method, as well as excellent intrinsic stability and catalytic activity.^18–21^ Research has shown that pure Ni and Fe oxides/hydroxides do not perform ideally for OER, while their compounds exhibit excellent OER activity.^21–23^ Corrigan observed that trace iron doping on nickel oxide electrodes significantly improved OER performance.^24^ Additionally, Tang *et al.* investigated the effect of Fe/Ni ratio on oxygen evolution performance by preparing NiFe-(oxy) hydroxides and found that appropriate guest metal substitution (Fe to Ni, Ni to Fe) effectively reduced Tafel slopes and overpotentials.^25^ Studies have shown that NiFe layered double metal hydroxides (NiFe-LDH) exhibit excellent OER performance under alkaline conditions.^26–28^ Regarding HER, the Volmer step still has a high kinetic barrier due to Fe^3+^ poor hydrogen adsorption capacity, limiting its hydrogen evolution rate.^29,30^ Therefore, it is crucial to further improve HER performance while optimizing and maintaining OER performance to achieve an efficient bifunctional catalyst for water splitting.

This study successfully prepared NiFe-LDH nanospheres on a nickel foam substrate using a hydrothermal synthesis method. By adjusting the concentration ratio of Fe^3+^ to Ni^2+^, the electrochemical performance of NiFe-LDH was influenced. In 1.0 M KOH electrolyte, efficient oxygen evolution reaction (OER) was achieved with an overpotential of 175 mV at a current density of 10 mA cm^−2^ and a Tafel slope of 80.32 mV dec^−1^. Simultaneously, the catalyst exhibited a hydrogen evolution overpotential of 185 mV and a Tafel slope of 120.61 mV dec^−1^ at −10 mA cm^−2^ current density. The material also demonstrated a low cell voltage of 1.52 V at 10 mA cm^−2^ and excellent stability after 12 hours of cycling. Due to the scarcity of freshwater resources and abundance of seawater, OER and HER performance were also tested in a 1.0 M KOH + seawater electrolyte environment. Direct hydrogen production by seawater electrolysis still faces numerous challenges. For the cathodic HER, the kinetics are relatively slow, necessitating electrocatalysts with high electrical conductivity. Another issue is that microorganisms and insoluble precipitates in natural seawater can form a layer over the active sites on the catalyst surface, hindering the progress of HER.^31^ While water electrolysis generates hydrogen at the cathode, it heavily relies on the efficiency and stability of OER at the anode. However, chloride ions (Cl^−^) in seawater may undergo chlorine oxidation reactions at the anode, and Cl^−^ can strongly corrode electrode and substrate materials. Unlike water electrolysis, direct seawater electrolysis faces challenges regarding the selectivity and stability of OER catalysts, especially considering that OER involves a four-electron transfer process. Although the oxygen evolution reaction (OER) is thermodynamically favorable, this inevitably leads to high energy barriers, resulting in slower kinetic rates during the seawater electrolysis process. Furthermore, due to the inevitable presence of chloride ions in seawater, Cl^−^ can strongly corrode electrode and substrate materials. The chloride evolution reaction (CER) also occurs at the anode at relatively high electrochemical potentials, competing with the oxygen evolution reaction (OER).^32^ The CER reaction primarily depends on factors such as pH, temperature, and potential.^33^ Depending on the pH value of the electrolyte, either chlorine evolution reaction (CER) or hypochlorite formation may occur at the anode. These reactions under acidic (CER) and alkaline (hypochlorite formation) conditions are represented by the following equations.^34^

1 In acidic media: 2Cl^−^ → Cl_2_ + 2e^−^ (*E*^0^ = 1.358 V *vs.* SHE)

2 In alkaline medium: Cl^−^ + 2OH^−^ → ClO^−^ + H_2_ + 2e^−^ (*E*^0^ = 1.57 V *vs.* SHE)

At room temperature, the Pourbaix diagram of an artificial seawater model^35^ indicates that OER is thermodynamically more favorable than CER. In an alkaline environment, hypochlorite formation becomes a critical issue to overcome; this reaction involves a two-electron transfer process and has superior kinetic characteristics compared to OER. However, from a thermodynamic perspective, OER is more favorable than hypochlorite formation. Moreover, unlike chlorine evolution reaction (CER), the standard potential for hypochlorite formation depends on the pH value of the solution and follows the potential trend of OER in the Pourbaix diagram. Therefore, there is an electrode potential difference of approximately 0.480 V between hypochlorite formation and OER.^36^ If the overpotential of the electrocatalyst is below this electrode potential, hypochlorite formation will not occur, preventing OER from competing with chlorine evolution reactions due to faster kinetics. However, in acidic conditions, the potential difference between CER and OER is smaller.^37^ Thus, under alkaline conditions, non-precious metal-based electrocatalysts are more suitable for seawater oxidation processes. Based on the dependence of hypochlorite formation on pH, the selection criteria for seawater electrolysis catalysts can be summarized as follows: the pH of the electrolyte should be maintained above 7.5, and the overpotential corresponding to the catalyst should be less than 480 mV. Specifically, when the pH is no less than 7.5, hypochlorous acid (HClO) formation precedes hypochlorite ion (ClO^−^) formation, while minimizing the potential difference between side reactions (such as chlorine evolution) and the oxygen evolution reaction (OER), thereby effectively inhibiting the occurrence of chlorine-related side reactions.^38^ Therefore, during alkaline seawater electrolysis, developing highly active OER catalysts is essential to ensure high selectivity, providing high current density at low overpotentials to prevent hypochlorite formation. The NiFe-LDH-4 catalyst exhibits a low overpotential of 230.5 mV@10 mA cm^−2^ and a Tafel slope of 36.18 mV dec^−1^ in alkaline seawater, with this overpotential being significantly lower than the range required for CER reactions (typically ≥480 mV), indicating that the catalyst favors OER over CER. The 12-hour cyclic stability test results of the NiFe-LDH-4 catalyst demonstrate no significant corrosion or performance degradation in alkaline seawater, indirectly indicating that the CER reaction does not dominate. Severe chlorine evolution would typically cause damage to the catalyst surface and structure. However, this material not only maintains its OER performance but also significantly enhances its HER performance. At a current density of −10 mA cm^−2^, it shows a hydrogen evolution overpotential of 96.5 mV and a Tafel slope of 66.97 mV dec^−1^. Compared to an alkaline electrolysis environment, the hydrogen evolution overpotential and Tafel slope have markedly decreased by 88.5 mV and 53.6 mV dec^−1^, respectively. In this process, ion migration and adsorption play a critical role; the large interlayer spacing of NiFe-LDH facilitates the diffusion of OH^−^ and Cl^−^ at active sites.^39,40^ Cl^−^ adsorption-induced structural modification, where Cl adsorption on Fe sites in seawater leads to the formation of chlorine-resistant active substances NiOOH and FeOOH, provides a rich Ni^3+^ surface for HER, optimizes H adsorption (Δ*G*_H↓_), and enhances H^+^ migration through hydrogen bonding networks, accelerating proton transfer, thereby directly reducing the HER Tafel slope.^41^ Finally, the sustained activity of the NiFe-LDH-4 catalyst was verified through high current density cyclic stability tests and SEM images after HER cycles in alkaline seawater conditions. To explore the potential of electrocatalysts for industrial applications, high current density testing was conducted under both alkaline and alkaline seawater environments. Under alkaline conditions, the NiFe-LDH-4 exhibited an OER overpotential of 476.3 mV and demonstrated excellent stability after 50 hours of cycling at a current density of 500 mA cm^−2^. An AEMWE device capable of producing hydrogen in an alkaline environment was constructed, with the NiFe-LDH-4@NF‖NiFe-LDH-4@NF electrolyzer showing outstanding stability. This indicates that the NiFe-LDH-4@NF electrocatalyst has significant industrial application potential for green hydrogen production.

## 2. Experiments

### 2.1 Material preparation

NiFe-LDH was prepared on nickel foam. All reagents were of analytical grade and used without further purification.

### 2.2 Pretreatment of nickel foam

First, cut nickel foam (5 × 5 cm^2^) was immersed in anhydrous ethanol and ultrasonically cleaned for 30 minutes. The foam was then immersed in deionized water for ultrasonic cleaning, alternating three times to remove surface oxide layers and impurities. After cleaning, the foam was placed in a vacuum drying oven at 60 °C for over 24 hours.

### 2.3 Synthesis of NiFe-LDH

NiFe-LDH was synthesized using a hydrothermal method with urea and ammonium fluoride as precipitants. Fe and Ni concentration ratios were 1 : 1, 2 : 1, 4 : 1, and 6 : 1. Ni(NO_3_)_2_·6H_2_O, 0.8 mmol Fe(NO_3_)_3_·9H_2_O, 16 mmol urea, and 8 mmol ammonium fluoride were dissolved in 60 mL deionized water and stirred for 40 minutes. The treated nickel foam was placed in a high-pressure reactor and heated in a constant temperature drying oven at 120 °C for 16 hours. After cooling to room temperature, the nickel foam was removed, rinsed with alcohol and deionized water at least three times, and dried at 60 °C for 8 hours. The synthesized products were named NiFe-LDH-*x* {*x* = 1/(1 : 1), *x* = 2/(2 : 1), *x* = 4/(4 : 1), *x* = 6/(6 : 1)} according to their concentration ratios. The following figure will also be named Fe : Ni=(1 : 1/1 : 2/1 : 4/1 : 6).

### 2.4 Material characterization

The crystal structure and phase purity of the samples were determined using X-ray diffraction (XRD, 7000, Shimadzu). Elemental composition and content were studied using an ESCALAB 250 X-ray photoelectron spectrometer (XPS) with an Al-Kα source. Sample morphology and structure were characterized using scanning electron microscopy (SEM, Gemini 300-71-31). Transmission electron microscopy (TEM) analysis was performed on an FEI Tecnai F20 TEM at 200 kV.

### 2.5 Electrochemical performance measurements

All electrocatalytic performances were tested on a CHI660E electrochemical workstation. A standard three-electrode system was used in 1 M KOH (pH = 13.7) and 1 M KOH seawater solution (pH = 13.51) electrolytes. The prepared samples served as working electrodes, Hg/HgO as the reference electrode, and Pt foil/carbon rod as counter electrodes for OER and HER, respectively. Cyclic voltammetry (CV), linear sweep voltammetry (LSV), electrochemical impedance spectroscopy (EIS), and cycling stability tests (*i*–*t*) were conducted. The linear sweep voltammetry (LSV) curve is calibrated with 90% IR compensation. The following formula was used to convert HER and OER voltages to RHE-related values: *E*_(*vs.* RHE)_ = *E*_(*vs.* Hg/HgO)_ + 0.059 × pH + 0.098 V. Additionally, overall water splitting performance was tested using a two-electrode system with a scan rate of 5 mV s^−1^.

## 3. Results and discussion

**Fig. 1:**
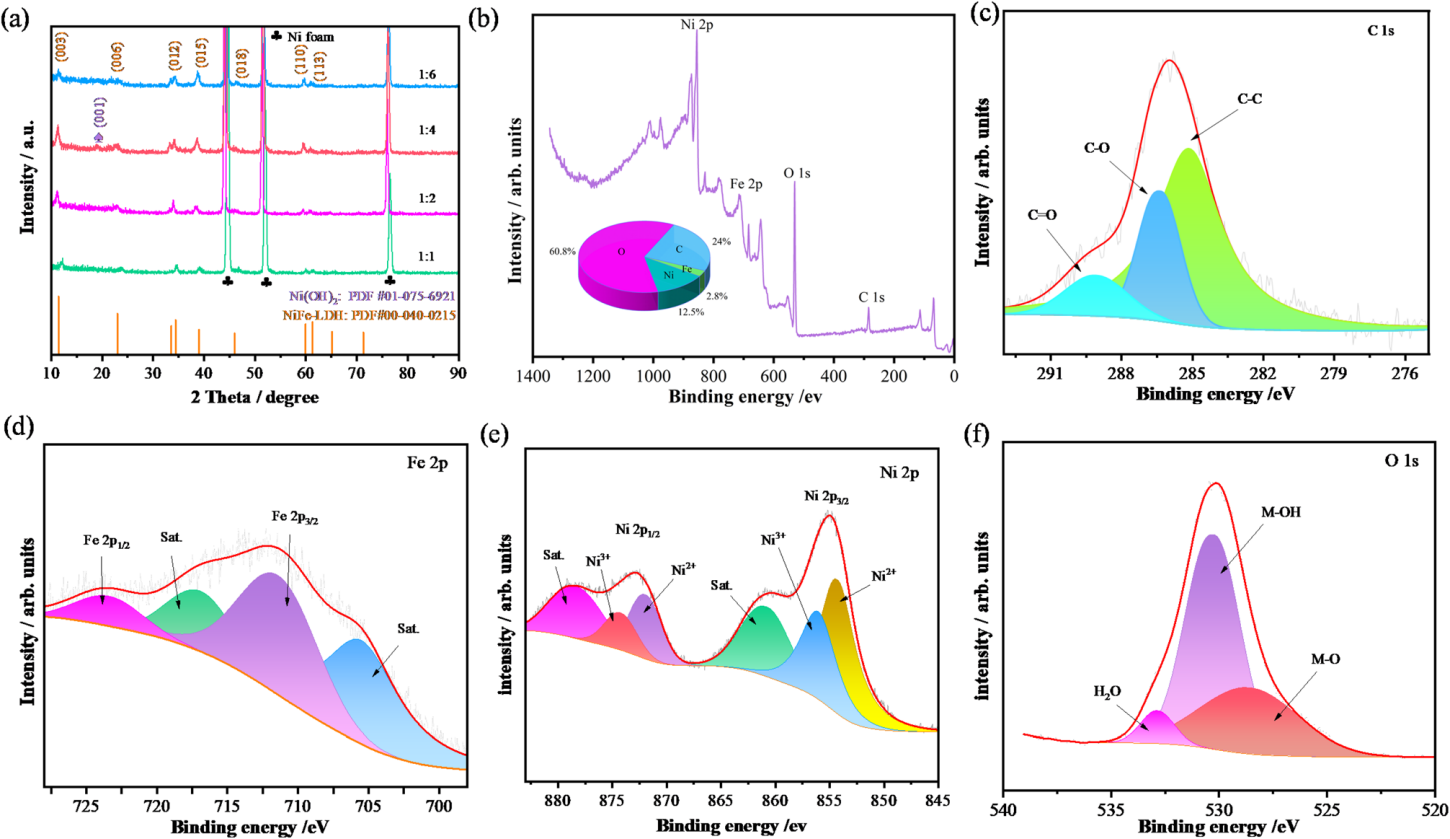
Structure characterization of as-fabricated samples (a) XRD patterns (b) full spectrum and percentage of each element (c) XPS of C 1s (d) Fe 2p (e) Ni 2p (f) O 1s.

The crystal structure characteristics of the prepared samples were first characterized by XRD, as shown in Fig. 1a. The three strong diffraction peaks originate from the nickel foam substrate. All samples exhibit characteristic peaks of layered double hydroxide compounds, which fully correspond to NiFe-LDH (JCPDS: 40-0215). The diffraction peaks at 2*θ* values of 11.4°, 23.0°, 34.4°, 39.0°, 59.9°, 61.2°, and 65.1° correspond to the (003), (006), (012), (015), (018), (110), and (113) crystal planes of NiFe-LDH, respectively. It can also be observed that the diffraction peak at approximately 19° corresponds to the (001) plane of the Ni(OH)_2_ phase (JCPDS: 01-075-6921). Its presence may be attributed to a byproduct formed during the material synthesis or surface oxidation process. The diffraction peak positions of the prepared samples are essentially consistent with those of NiFe-LDH, indicating the successful growth of NiFe-LDH nanophase on the nickel foam substrate *via* hydrothermal synthesis. XPS analysis was conducted on the prepared NiFe-LDH-4 sample to examine its chemical composition, elemental valence states, and binding energies. Fig. 1b shows the full spectrum of the sample, revealing four signal peaks corresponding to Fe, Ni, O, and C, confirming the presence of these elements. The elemental content analysis shows that the percentages of Fe, Ni, O, and C in the NiFe-LDH-4 sample are 2.8%, 12.5%, 24%, and 60.8%, respectively. All element peak positions were calibrated using the C 1s peak (284.8 eV) as a reference (Fig. 1c). The Fe 2p spectrum (Fig. 1d) can be fitted into four different spectral peaks, with binding energies at 712.05 eV and 723.9 eV attributed to Fe 2p_3/2_ and Fe 2p_1/2_ characteristic peaks, respectively. The peaks at 705.09 eV and 717.45 eV are two satellite peaks, confirming Fe^3+^ as the primary existing form.^42^ The Ni 2p spectrum (Fig. 1e) can be fitted into six different spectral peaks, with binding energies at 861.25 eV and 878.85 eV corresponding to two satellite peaks. The two higher broad peaks correspond to Ni 2p_3/2_ and Ni 2p_1/2_, both containing Ni^3+^ and Ni^2+^, indicating that Ni exists mainly in the form of coexisting Ni^3+^ and Ni^2+^ in the sample.^43^ The O 1s spectrum (Fig. 1f) shows that the peaks at binding energies of 528.8 eV, 530.35 eV, and 532.9 eV can be assigned to M–O bonds, hydroxides (M–OH), and water, respectively.

**Fig. 2:**
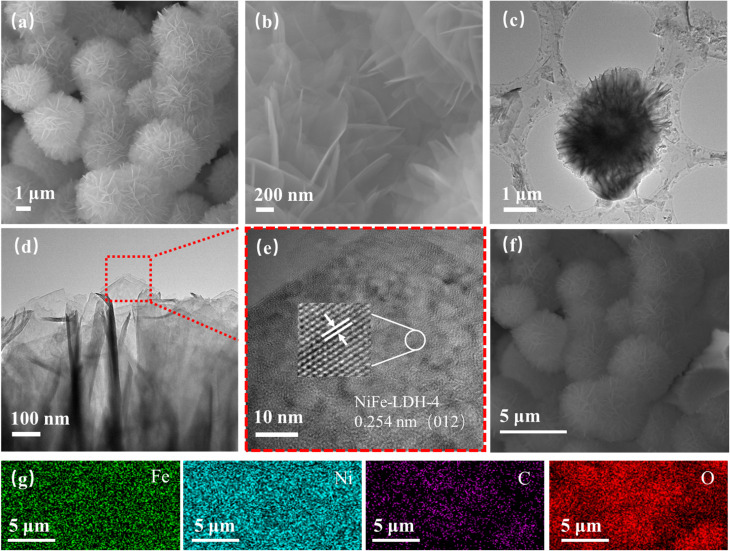
Morphology and structural characteristics of the prepared sample (a and b) SEM images of NiFe-LDH-4 (c–e) TEM images of NiFe-LDH-4 samples (f and g) elemental mapping of NiFe-LDH-4 samples.

The morphology and structure of NiFe-LDH samples prepared under different concentration conditions were characterized by scanning electron microscopy (SEM). From the microscopic morphology images of the prepared NiFe-LDH samples in Fig. 2(a and b) and S1(a–d),† it can be clearly seen that the outer surface of all four concentration ratios of NiFe-LDH samples exhibits a uniformly grown spherical structure. Spherical NiFe-LDH with a diameter of only a few micrometers uniformly covers the nickel foam substrate, This is because many nanosheets are interconnected to form NiFe LDH nanospheres. These nanosheets are intertwined, providing a large specific surface area for the NiFe-LDH electrocatalyst and exposing a large number of active sites. The increase in active sites further accelerates the reaction rate, enabling the electrocatalyst to exhibit excellent catalytic activity. Comparison of the morphologies of the four concentrations shows that NiFe-LDH-4 nanospheres are more uniformly distributed, with higher sheet density, larger specific surface area, and more active sites. The microstructural characteristics of the NiFe-LDH-4 sample were further characterized by transmission electron microscopy (TEM). Fig. 2c shows that the sample morphology is nanospherical, consistent with the SEM results. The HRTEM morphology in Fig. 2(d and e) reveals lattice fringes with an interplanar spacing of 0.254 nm, corresponding to the (012) crystal plane of the NiFe-LDH-4 sample. Fig. 2(f and g) shows the energy dispersive spectroscopy (EDS) map of the NiFe-LDH-4 sample, indicating that Fe, Ni, C, and O elements are uniformly distributed throughout the nanosphere, further confirming the successful preparation of the NiFe-LDH-4 sample.

**Fig. 3:**
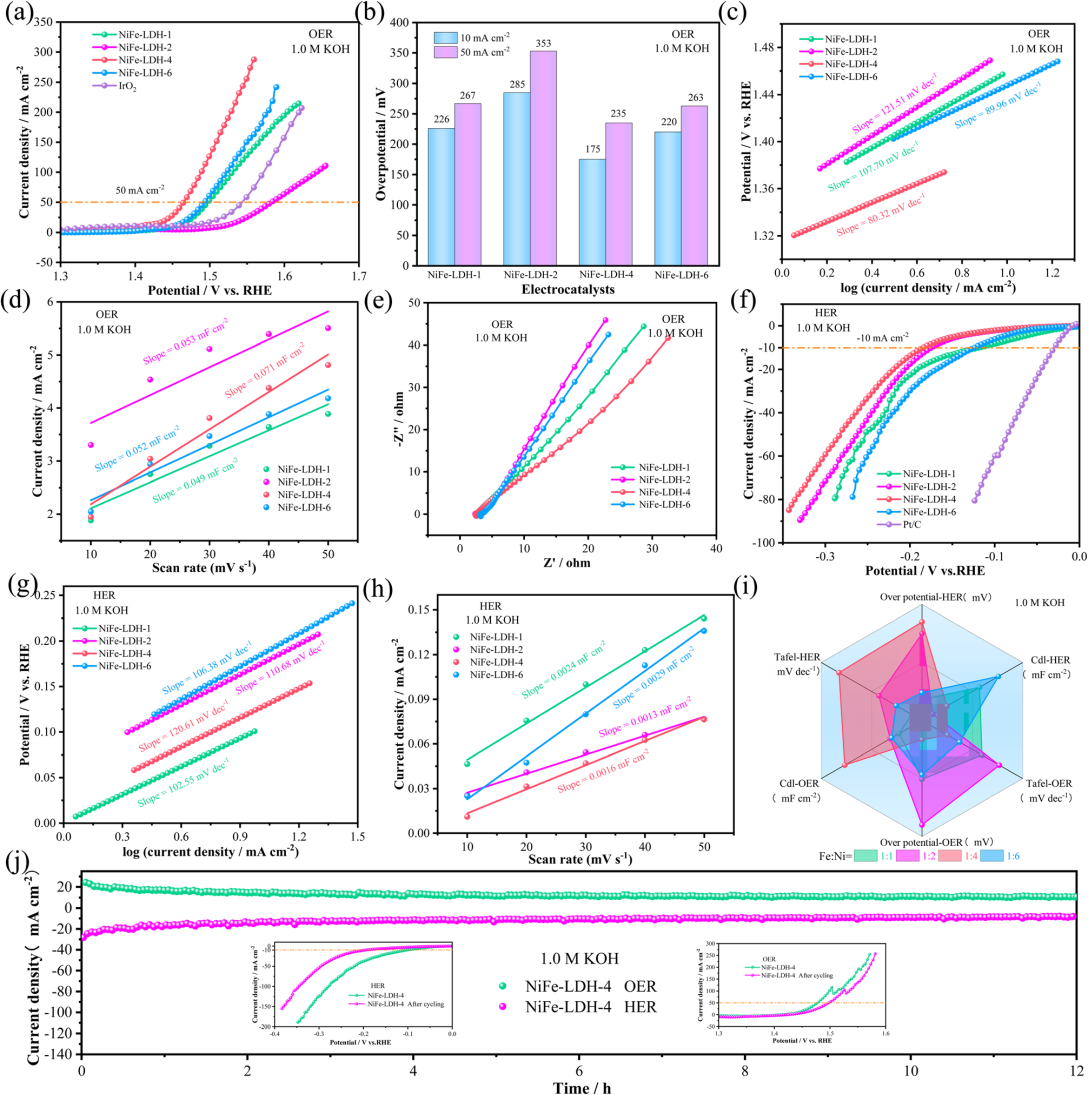
OER and HER performance (a) polarization curve at a scanning rate of 5 mV s^−1^ (b) overpotential bar chart (c) Tafel Plot (d) CV curve of double layer capacitor (*C*_dl_) (e) Nyquist plot (f) polarization curve at a scanning rate of 5 mV s^−1^ (g) Tafel Plot (h) CV curve of double layer capacitor (*C*_dl_) (i) radar plot (j) timing ampere stability test illustration: LSV curve before and after cycles.

To investigate the electrocatalytic performance of the prepared NiFe-LDH samples, their OER performance in alkaline electrolyte (1.0 M KOH) was first studied. For comparison, the catalytic performance of commercial IrO_2_ catalyst was also measured. Fig. 3a shows that NiFe-LDH-4 has a lower overpotential than the noble metal IrO_2_ at a current density of 50 mA cm^−2^, indicating superior electrocatalytic performance. Fig. 3(a and b) show that NiFe-LDH-4 has the lowest overpotential, only 175 mV at a current density of 10 mA cm^−2^, which is lower than the overpotentials of NiFe-LDH-1 (226 mV@10 mA cm^−2^), NiFe-LDH-2 (285 mV@10 mA cm^−2^), and NiFe-LDH-6 (220 mV@10 mA cm^−2^) catalysts, indicating that NiFe-LDH-4 has the best catalytic activity. To further investigate the reaction kinetics of the catalysts, the corresponding Tafel slopes were obtained from the LSV curves, as shown in Fig. 3c. The Tafel slope of the NiFe-LDH-4 catalyst (80.32 mV dec^−1^) is smaller than those of NiFe-LDH-1 (107.70 mV dec^−1^), NiFe-LDH-2 (121.51 mV dec^−1^), and NiFe-LDH-6 (89.96 mV dec^−1^), indicating that the NiFe-LDH-4 sample has rapid charge transfer capability and kinetic reaction rate. Moreover, the electrochemically active surface area (ECSA) is an important indicator for evaluating catalyst activity, representing the effective area involved in the catalytic reaction. Using the proportional relationship between ECSA and double-layer capacitance (*C*_dl_), the *C*_dl_ value can be used as a measure of electrocatalytic activity. And the *C*_dl_ value was obtained based on its CV plot S2(a–d).† As shown in Fig. 3d, the *C*_dl_ value of NiFe-LDH-4 (0.071 mF cm^−2^) is higher than those of NiFe-LDH-1 (0.049 mF cm^−2^), NiFe-LDH-2 (0.053 mF cm^−2^), and NiFe-LDH-6 (0.052 mF cm^−2^). Electrochemical impedance spectroscopy (EIS) also plays an important role in catalyst kinetics. In Fig. 3e, the linear segment in the low-frequency region corresponds to the diffusion resistance of electrolyte ions, the intercept on the real axis in the high-frequency region represents the solution resistance (*R*_s_), and the diameter of the semicircular arc reflects the charge transfer resistance (*R*_ct_). To further analyze the electrochemical behavior, an equivalent circuit fitting was conducted on the high-frequency region of the EIS curve in Fig. 3e, and the results are presented in Fig. S3a and Table S1.† The fitting yielded the corresponding *R*_ct_ and *R*_s_ values. In the EIS plot, a smaller arc radius indicates lower charge transfer impedance, which implies a smaller *R*_ct_ value and faster reaction kinetics. The *R*_ct_ value mainly reflects the efficiency of charge transfer at the catalyst–electrolyte interface; a lower *R*_ct_ suggests more efficient charge transport and thus enhanced catalytic activity. Among the samples, NiFe-LDH-4 exhibits the lowest *R*_ct_ value (1.15 Ω cm^−2^) and *R*_s_ value (2.518 Ω), indicating superior electrical conductivity and efficient charge transport properties, which significantly improve its catalytic performance. It can be observed that NiFe-LDH-4 exhibits higher overpotential, Tafel slope, and *C*_dl_ values compared to other composition ratios, indicating superior intrinsic activity. This is consistent with the earlier analysis, further demonstrating that NiFe-LDH-4 possesses excellent electrochemical performance and catalytic activity. We also studied the Bote diagram S2(e, f),† and found that as the voltage increases, the electron transfer rate also increases. This indicates that the catalyst has a high electron transfer rate and high catalytic activity.

Secondly, the HER performance of the samples in alkaline electrolyte (1.0 M KOH) was also investigated. Fig. 3f shows that the HER overpotential of the noble metal Pt/C catalyst at −10 mA cm^−2^ is 44.2 mV, which is lower than NiFe-LDH-1 (118 mV@−10 mA cm^−2^), NiFe-LDH-2 (175 mV@−10 mA cm^−2^), NiFe-LDH-4 (185 mV@−10 mA cm^−2^), and NiFe-LDH-6 (124 mV@−10 mA cm^−2^). The Tafel slope values in Fig. 3g are: NiFe-LDH-1 (102.55 mV dec^−1^) < NiFe-LDH-6 (106.38 mV dec^−1^) < NiFe-LDH-2 (110.68 mV dec^−1^) < NiFe-LDH-4 (120.61 mV dec^−1^). The *C*_dl_ values in Fig. 3h show that NiFe-LDH-6 (0.0029 mF cm^−2^) is higher than NiFe-LDH-1 (0.0024 mF cm^−2^), NiFe-LDH-2 (0.0013 mF cm^−2^), and NiFe-LDH-4 (0.0016 mF cm^−2^). In summary, NiFe-LDH-1 exhibits better HER catalytic activity, NiFe-LDH-6 has a slightly better electrochemically active surface area, while NiFe-LDH-4's HER catalytic performance is not ideal, providing a foundation and direction for future work on improving the HER performance of NiFe-LDH-4. Fig. 3i is a radar chart of different concentration ratios, which more intuitively reflects OER and HER performance, including LSV, Tafel, and *C*_dl_ values. It can be seen that NiFe-LDH-4 has the most excellent OER performance, far surpassing other samples. The cycling stability of the catalyst is one of the important indicators for evaluating material performance. The durability of the prepared catalysts was assessed by chronopotentiometry, as shown in Fig. 3j. The two lines in the figure represent the cycling stability curves of OER and HER, respectively. The inset shows the LSV curves before and after cycling, indicating that the prepared electrocatalyst has good cycling stability. Table 1 summarizes the OER and HER performance of several previously reported LDH-based electrocatalysts.^44–49^ Under a current density of 10 mA cm^−2^ in 1.0 M KOH electrolyte, most of these catalysts exhibit excellent OER and HER activities. However, compared to the other catalysts listed, the electrocatalyst prepared in this study demonstrates superior OER performance, while its HER activity remains comparable.

**Table 1:** Electrocatalytic performances of various catalysts

Materials	OER (mV)	HER (mV)	Electrolyte	Ref.
NiCo-LDH	303@50 mA cm^−2^	227	1.0 M KOH	44
NiSe_2_@Fe-NiCo-LDH	260	130	1.0 M KOH	45
CoMo-LDH	290	115	1.0 M KOH	46
Ru-CoV-LDH	230	230	1.0 M KOH	47
NiFeAu-LDH	181	89	1.0 M KOH	48
Co_3_O_4_@NiFe-LDH	215	79	1.0 M KOH	49
NiFe-LDH	175	185	1.0 M KOH	This study

**Fig. 4:**
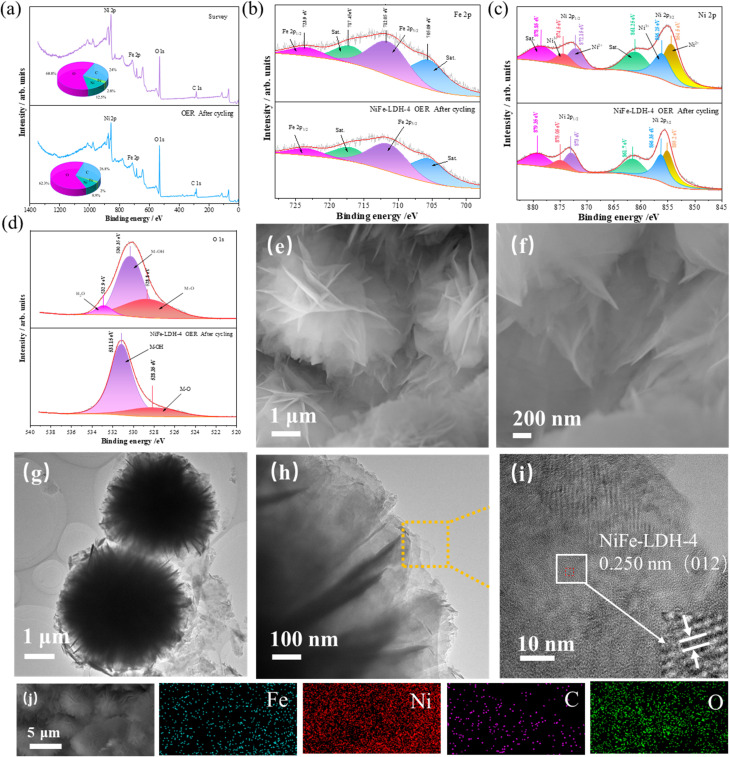
XPS, SEM, and TEM images after cycling stability test (a) proportion of each element before and after cycling and full spectrum (b) Fe 2p (c) Ni 2p (d) O 1s (e and f) SEM images of NiFe-LDH-4 (g–i) TEM images of NiFe-LDH-4 samples (j) elemental mapping of NiFe-LDH-4 samples.

The study of its surface structure is a prerequisite for in-depth understanding of the OER mechanism. To compare the changes in the electronic structure of the catalyst surface before and after cycling, XPS measurements were performed on the samples after OER cycling to study the surface chemical state of the catalyst. Fig. 4a shows the XPS full spectrum and elemental content of the NiFe-LDH-4 catalyst before and after cycling. The full spectrum confirms the presence of Fe, Ni, C, and O. The content chart shows that the content of C and O increased by 2.8% and 1.5%, respectively, while the content of Ni and Fe decreased by 3.6% and 0.8%, respectively, due to the corresponding electrochemical reactions occurring on the surface of the electrode material. Fig. 4b shows the Fe 2p spectra before and after OER cycling. All peaks remained essentially unchanged after cycling, with only a slight decrease in the area of the Fe 2p_3/2_ orbital, highlighting the stability of the catalyst's surface structure. The Ni 2p spectra in Fig. 4c show that after OER cycling, all peaks shifted to higher energy regions compared to before cycling. The peak values of Ni^3+^ and Ni^2+^ in the Ni 2p_3/2_ orbital shifted by 0.01 eV and 0.7 eV, respectively. The peak values of Ni^3+^ and Ni^2+^ in the Ni 2p_1/2_ orbital shifted by 0.55 eV and 0.85 eV, respectively. The two satellite peaks shifted by 0.45 eV and 0.5 eV. The Ni^3+^/Ni^2+^ peak area ratio in the Ni 2p_3/2_ orbital increased after OER cycling. According to research, a higher proportion of Ni^3+^ is beneficial for enhancing catalytic activity during the OER process, as Ni^3+^ is closely related to NiOOH, which is an important active substance in OER.^50^ The O 1s spectrum (Fig. 4d) shows an increase in the M–OH peak content after cycling and a positive shift of 0.8 eV, while the M–O peak value shifted by 0.45 eV. During this OER cycling process, a dynamic transformation from M–O to M–OH was achieved, leading to an increase in M–OH content and the disappearance of adsorbed water. From the above XPS spectra, it was found that after the OER cycling process, all peak shifts were small or did not occur, confirming the structural stability of the sample. To further confirm the good structural stability of the prepared samples, SEM was used to characterize the structure of the samples after OER cycling, as shown in Fig. 4(e and f). It can be seen that after 12 hours of cycling, there were no significant changes in the structural characteristics of the samples, except for some nanosheets in the nanospheres becoming sparse, mainly due to a small amount of material falling off the nickel foam after long-term immersion in potassium hydroxide. The TEM image after cycling (Fig. 4g) shows that the NiFe-LDH sample still maintains its nanosphere morphology, indicating that the NiFe-LDH-4 catalyst has high structural stability. The HRTEM morphology in Fig. 4(h and i) shows lattice fringes with an interplanar spacing of 0.25 nm, corresponding to the (012) crystal plane of the NiFe-LDH sample. The lattice spacing of the sample after cycling decreased by 0.004 nm, almost unchanged, once again highlighting the structural stability of the sample. Fig. 4j shows the EDS map of the NiFe-LDH-4 sample after cycling, which similarly proves that Fe, Ni, C, and O are uniformly distributed throughout the nanosphere.

In summary, the above experiments successfully prepared the NiFe-LDH catalyst through a simple one-step hydrothermal synthesis method. The prepared NiFe-LDH-4 sample exhibited excellent electrocatalytic performance in a 1.0 M KOH alkaline electrolyte environment. At a current density of 10 mA cm^−2^, its oxygen evolution overpotential was only 175 mV, and at 50 mA cm^−2^, the overpotential was merely 235 mV, with a corresponding Tafel slope of 80.32 mV dec^−1^. Moreover, this catalyst maintained cycling stability for 12 hours. Water electrolysis for hydrogen production is considered a highly promising green energy technology.^51–53^ Currently, domestic and international research in this area primarily focuses on alkaline freshwater electrolysis. This undoubtedly exacerbates the global shortage and uneven distribution of freshwater resources. In contrast to the scarcity of freshwater resources, seawater represents approximately 96.5% of the world's total renewable energy reserves,^54–56^ offering abundant resources. Therefore, seawater electrolysis is a potentially scalable hydrogen production technology.

**Fig. 5:**
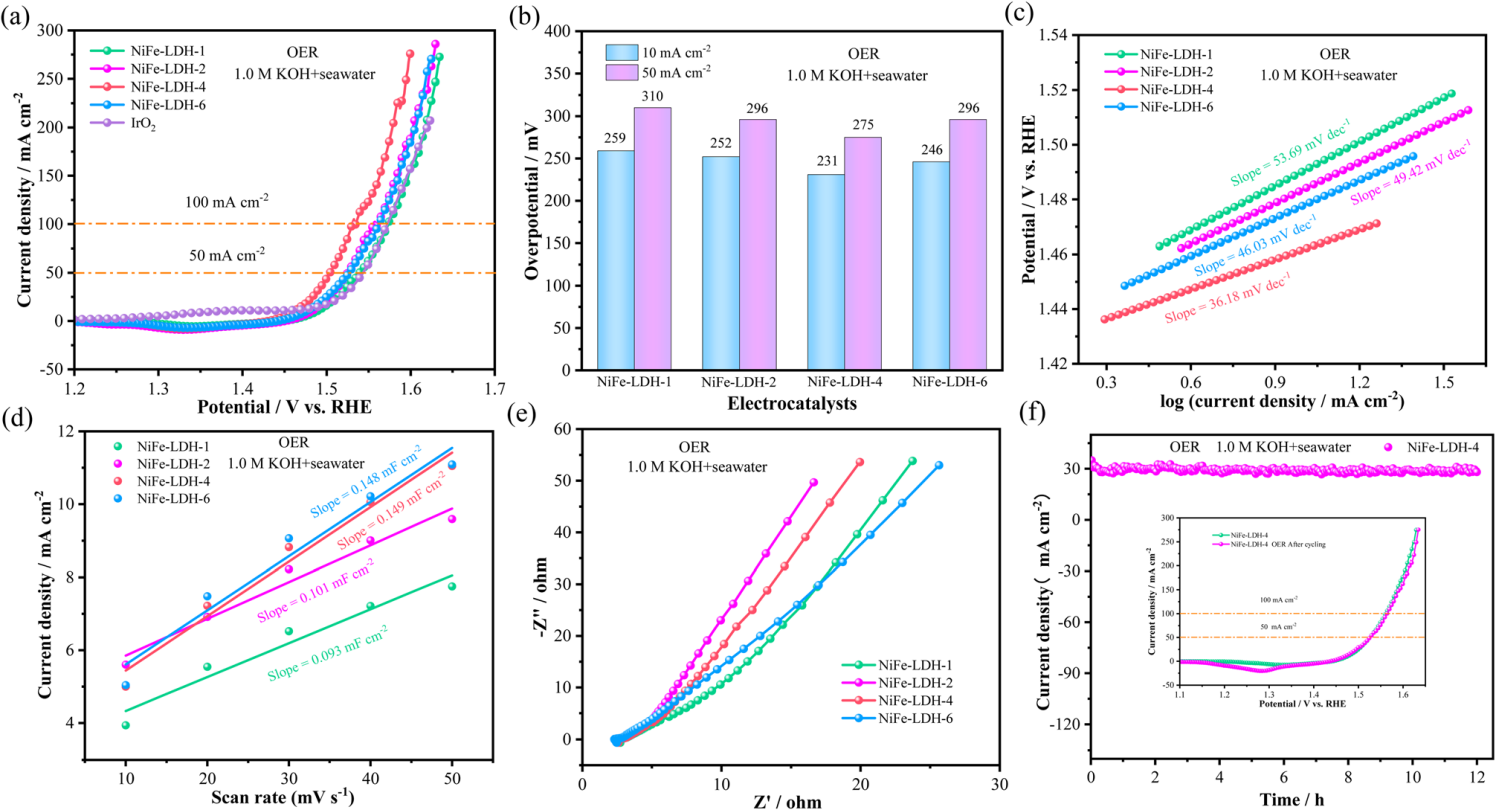
OER performance in seawater (a) polarization curves at scan rate of 5 mV s^−1^ (b) bar chart (c) Tafel plots (d) CV curves of double-layer capacitance (*C*_dl_) (e) Nyquist plots (f) chronoamperometric stability tests the insets is LSV curves before ang after cycle.

The study then investigated the HER and OER performance of the NiFe-LDH catalyst in alkaline seawater (1.0 M KOH + seawater) with a pH of 13.51. First, the OER performance of the NiFe-LDH catalyst was examined. The LSV curves (Fig. 5a) show that the NiFe-LDH-4 sample has lower overpotentials than the noble metal IrO_2_ at current densities of 50 mA cm^−2^ and 100 mA cm^−2^. The bar graph of overpotentials at different current densities (Fig. 5b) shows that NiFe-LDH-4 (231 mV/275 mV@10/50 mA cm^−2^) is lower than NiFe-LDH-1 (259 mV/310 mV@10/50 mA cm^−2^), NiFe-LDH-2 (252 mV/296 mV@10/50 mA cm^−2^), and NiFe-LDH-6 (246 mV/296 mV@10/50 mA cm^−2^). The corresponding Tafel slopes (Fig. 5c) indicate that these catalysts have rapid reaction kinetics and good charge transfer characteristics. The Tafel slope of the NiFe-LDH-4 catalyst (36.18 mV dec^−1^) is smaller than NiFe-LDH-1 (53.69 mV dec^−1^), NiFe-LDH-2 (49.42 mV dec^−1^), and NiFe-LDH-6 (46.03 mV dec^−1^). To further verify the intrinsic activity of the catalysts, their *C*_dl_ values were examined (Fig. 5d): NiFe-LDH-4 (0.149 mF cm^−2^) > NiFe-LDH-6 (0.148 mF cm^−2^) > NiFe-LDH-2 (0.101 mF cm^−2^) > NiFe-LDH-1 (0.093 mF cm^−2^). Fig. 5e shows the EIS test results for the four samples, with NiFe-LDH-2 having the largest slope in the linear region, followed by NiFe-LDH-4. An equivalent circuit fitting has been added for the EIS plot in Fig. 5e, and the results are presented in Fig. S3b and Table S2.† The charge transfer resistance (*R*_ct_) of the NiFe-LDH-4 electrocatalyst is 0.454 Ω cm^−2^, which is lower than that of NiFe-LDH-2 (0.461 Ω cm^−2^), NiFe-LDH-6 (0.473 Ω cm^−2^), and another NiFe-LDH-6 sample (0.586 Ω cm^−2^). These results indicate that NiFe-LDH-4 exhibits a lower charge transfer resistance, suggesting improved charge transport capability and enhanced catalytic activity. In summary, NiFe-LDH-4 demonstrates excellent OER performance. Cycling stability is particularly important for evaluating catalyst performance. Fig. 5f shows that the NiFe-LDH-4 sample maintains excellent voltage stability after 12 hours of cycling. Moreover, the corresponding LSV curves indicate that the overpotentials at current densities of 50 mA cm^−2^ and 100 mA cm^−2^ remain almost unchanged before and after cycling, demonstrating the excellent structural stability of the prepared samples.

**Fig. 6:**
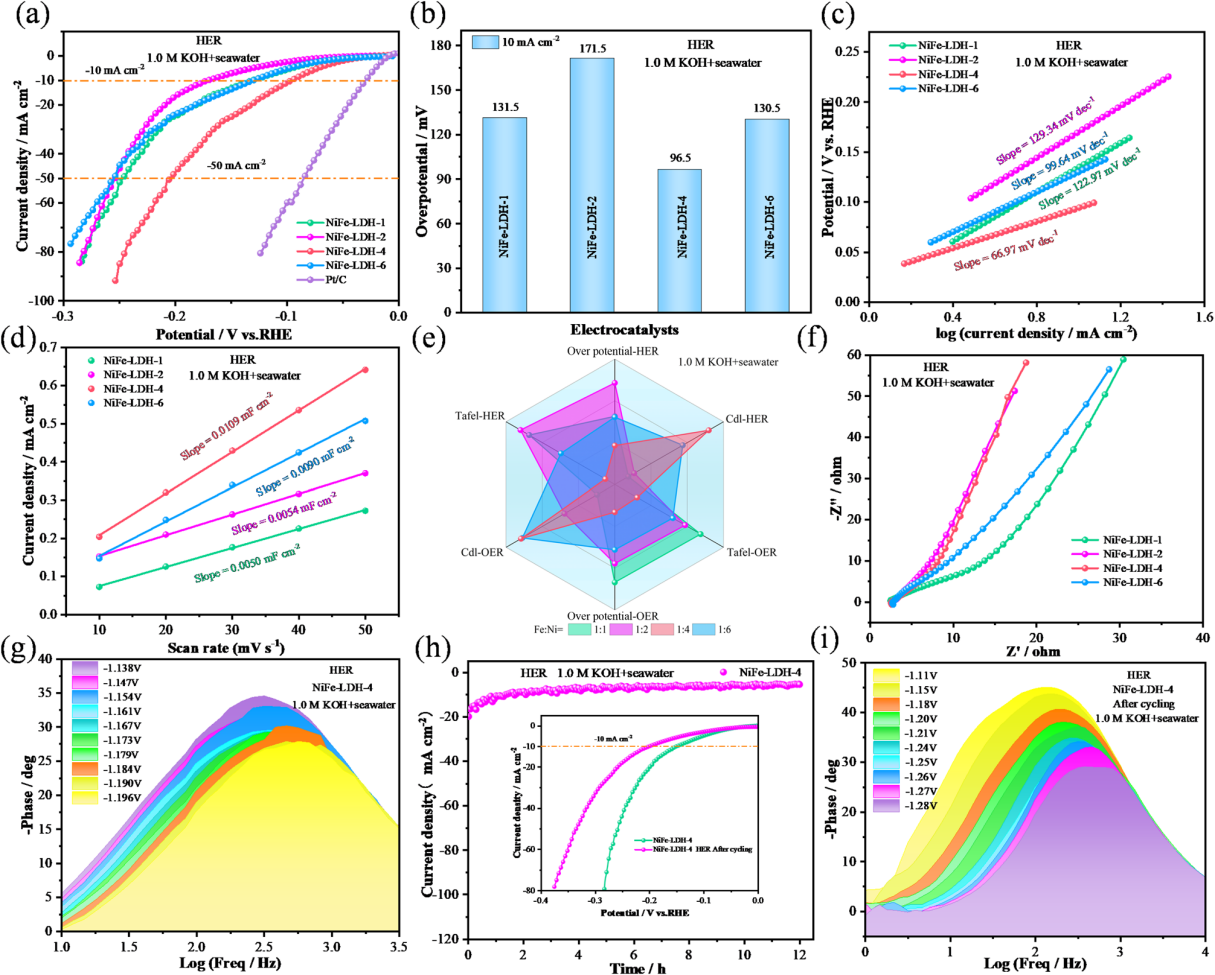
HER performance in seawater (a) polarization curve at a scanning rate of 5 mV s^−1^ (b) bar chart (c) Tafel plot (d) CV curve of double-layer capacitor (*C*_dl_) (e) HER and OER radar plot (f) Nyquist plot (g) Bode plot of NiFe-LDH-4 sample before cycling at multiple voltages (h) timing current stability test illustration LSV curve before and after cycling (i) Bode plot of NiFe-LDH-4 sample after cycling at multiple voltages.

The study then examined the HER performance of the NiFe-LDH catalyst. As shown in Fig. 6a, the LSV curves of the samples were compared with that of the noble metal Pt/C catalyst. Fig. 6(a and b) show that at a current density of 10 mA cm^−2^, except for the noble metal Pt/C catalyst with an HER overpotential of only 44.2 mV, the HER overpotential of NiFe-LDH-4 (96.5 mV) is significantly lower than NiFe-LDH-1 (131.5 mV), NiFe-LDH-2 (171.5 mV), and NiFe-LDH-6 (130.5 mV). To investigate the catalytic reaction kinetics, Tafel slopes were calculated from the LSV curves (Fig. 6c). NiFe-LDH-4 has the smallest Tafel slope (66.97 mV dec^−1^), lower than NiFe-LDH-1 (122.97 mV dec^−1^), NiFe-LDH-2 (129.34 mV dec^−1^), and NiFe-LDH-6 (99.64 mV dec^−1^). The electrochemically active surface area (ECSA) was used to study the origin of catalyst activity, and the corresponding *C*_dl_ values were measured (Fig. 6d). The *C*_dl_ value of NiFe-LDH-4 (0.011 mF cm^−2^) is higher than NiFe-LDH-1 (0.005 mF cm^−2^), NiFe-LDH-2 (0.005 mF cm^−2^), and NiFe-LDH-6 (0.009 mF cm^−2^), indicating that NiFe-LDH-4 has a large number of active centers. The radar chart in Fig. 6e reflects three important parameters (LSV, Tafel, and *C*_dl_) for OER and HER performance of the samples in an alkaline seawater environment, demonstrating that NiFe-LDH-4 has the best overall performance. To evaluate the ion diffusion capability, electrochemical impedance spectroscopy (EIS) measurements were carried out (Fig. 6f). A steeper slope in the linear region of the EIS plot at low frequencies indicates lower ion diffusion resistance. NiFe-LDH-4 exhibits the largest slope in this region, suggesting that it possesses the lowest ion diffusion resistance and intrinsic resistance among the samples. In addition, an equivalent circuit fitting was performed on the EIS curve in Fig. 6f, and the results are shown in Fig. S3c and Table S3.† The fitted charge transfer resistance (*R*_ct_) and solution resistance (*R*_s_) of the NiFe-LDH-4 electrocatalyst are 7.093 Ω cm^−2^ and 2.784 Ω, respectively, which are the lowest among all the samples. These results indicate that NiFe-LDH-4 exhibits excellent HER performance in seawater. The Bode plot (Fig. 6g) represents the phase angle–frequency curve reflecting the catalyst's kinetic characteristics.^57^ To evaluate the reaction kinetics, the charge transfer time constant (*τ*) was calculated. The time constant (*τ*) can be determined from the frequency at which the phase angle reaches its peak (*f*_peak_) in the Bode plot, using the formula:3
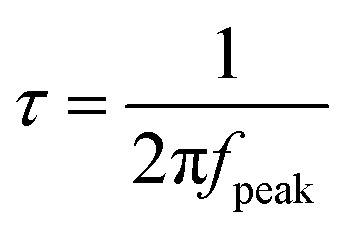


A smaller time constant (*τ*) indicates a faster charge transfer rate and superior catalytic kinetics. The kinetic behavior of the NiFe-LDH-4 catalyst before cycling (Fig. 6g) was analyzed under different applied potentials. The results show that as the applied voltage increases (*i.e.*, the overpotential increases, from −1.138 V to −1.196 V), the peak of the phase angle in the Bode plot shifts toward higher frequency regions. At −1.138 V, the peak frequency (*f*_peak_) is approximately 316 Hz, corresponding to a calculated time constant *τ* of about 0.50 ms. When the voltage increases to −1.196 V, the *f*_peak_ rises to about 501 Hz, and the time constant *τ* decreases to approximately 0.32 ms. The significant decrease in the time constant quantitatively demonstrates that the charge transfer process at the catalyst surface accelerates notably with increasing overpotential. The constant current test (Fig. 6h) verifies the good cycling stability of the prepared electrocatalyst, with the inset showing a comparison of LSV curves before and after cycling. For the NiFe-LDH-4 catalyst after cycling (Fig. 6i), the Bode plot was analyzed following a long-term HER stability test to evaluate its stability. The results indicate that the time constant remains at a very low level. Under an applied voltage of −1.20 V, the calculated time constant *τ* is approximately 1.0 ms. These findings suggest that, even after prolonged operation, the NiFe-LDH-4 electrode material still maintains efficient electron transfer capability and excellent electrocatalytic performance.

**Fig. 7:**
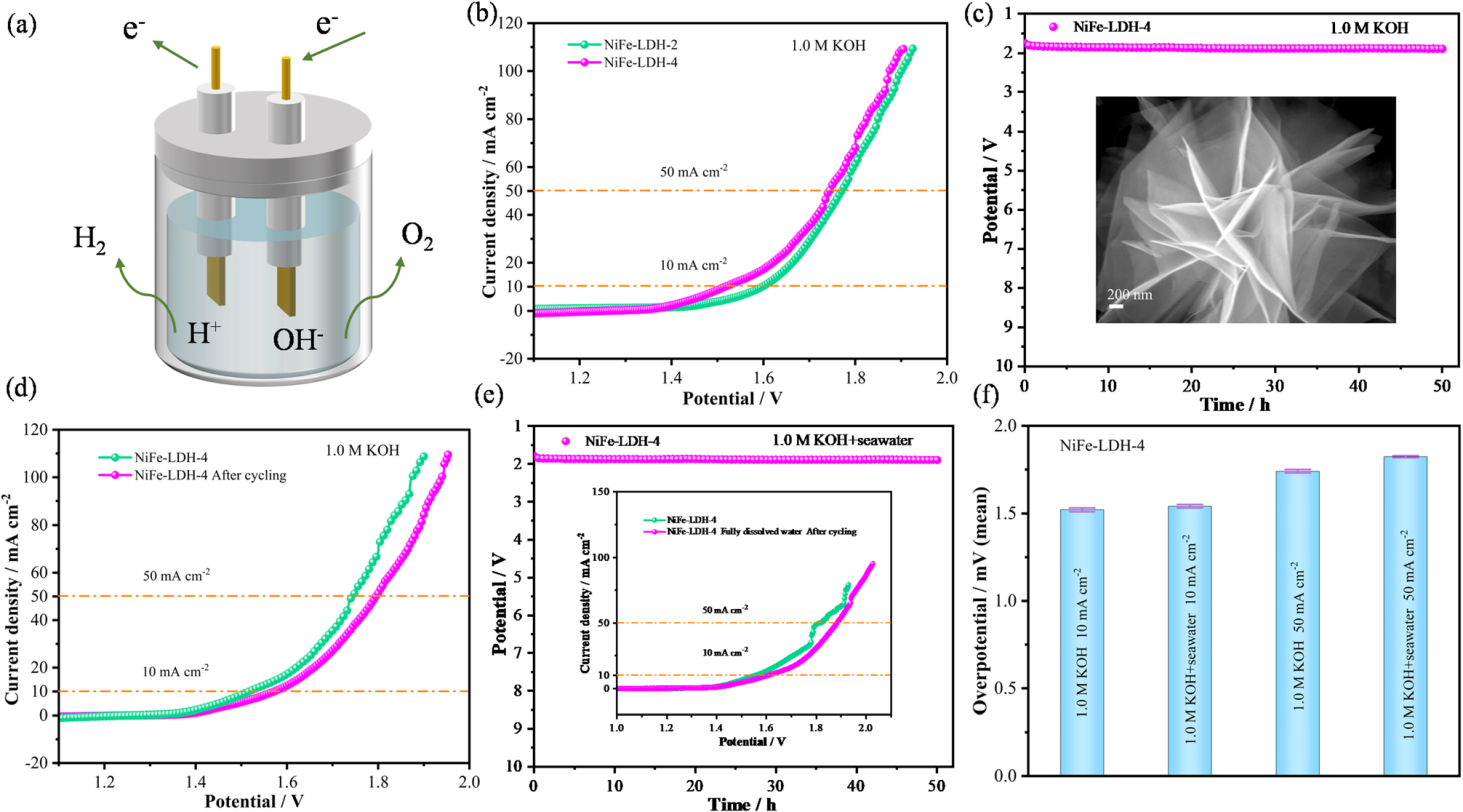
Shows the overall water splitting performance of the electrocatalyst. (a) Schematic diagram of the overall water splitting device. (b) LSV curve in 1.0 M KOH. (c) Illustration of chronoamperometric stability test. SEM image of NiFe-LDH-4. (d) LSV before and after cycling in 1.0 M KOH. (e) The illustration of the chronoamperometric stability curve in alkaline seawater is the LSV curve (f) of NiFe-LDH-4 and the voltage comparison chart.

To investigate the potential of electrocatalysts in water splitting, their overall performance was evaluated using a two-electrode system, as illustrated in Fig. 7a. The catalyst samples were used as both anode and cathode materials simultaneously. During the cathode reaction, OH^−^ is converted to O_2_, while H_2_ is generated at the anode. Electrochemical measurements were carried out in both 1.0 M KOH and alkaline seawater electrolytes. First, all LSV curves have been corrected using 90% iR compensation. In 1.0 M KOH, the LSV curves of the optimal samples, NiFe-LDH-4 and NiFe-LDH-2, are presented in Fig. 7b. At current densities of 10 mA cm^−2^ and 50 mA cm^−2^, the NiFe-LDH-4 catalyst exhibited overall water-splitting voltages of 1.52 V and 1.74 V, respectively, lower than those of NiFe-LDH-2 (1.60 V and 1.77 V). Cycle stability is another crucial factor in evaluating the performance of the samples. Fig. 7c shows the potentiostatic stability test results of the NiFe-LDH-4 catalyst. After a 50-hour cycling stability test, its voltage value exhibits only minor fluctuations. Fig. 7d displays the LSV curves before and after water-splitting cycling, showing minimal differences. At a current density of 10 mA cm^−2^, the overpotential shows only a 0.05 V difference, indicating the catalyst's excellent structural stability. Fig. 7e presents the potentiostatic stability test results of the NiFe-LDH-4 catalyst in seawater. The voltage remains nearly constant over a 50-hour cycling test, demonstrating outstanding long-term stability in real seawater environments. The inset displays the LSV curves before and after cycling. At 10 mA cm^−2^, the voltage remains nearly constant, emphasizing the material's stability. The bar chart in Fig. 7f displays the water-splitting voltages of NiFe-LDH-4 at two different environments and current densities. In 1.0 M KOH, the output voltages were 1.52 V@10 mA cm^−2^ and 1.74 V@50 mA cm^−2^. In 1.0 M KOH with seawater electrolyte, the output voltages were 1.54 V@10 mA cm^−2^ and 1.82 V@50 mA cm^−2^. In addition, error bars have been added to Fig. S5† and 7d to reflect the variation range of the experimental data. The results show that the errors are small, further confirming the reproducibility and reliability of the experimental data. In summary, the catalyst demonstrates excellent water-splitting performance under both conditions.

**Fig. 8:**
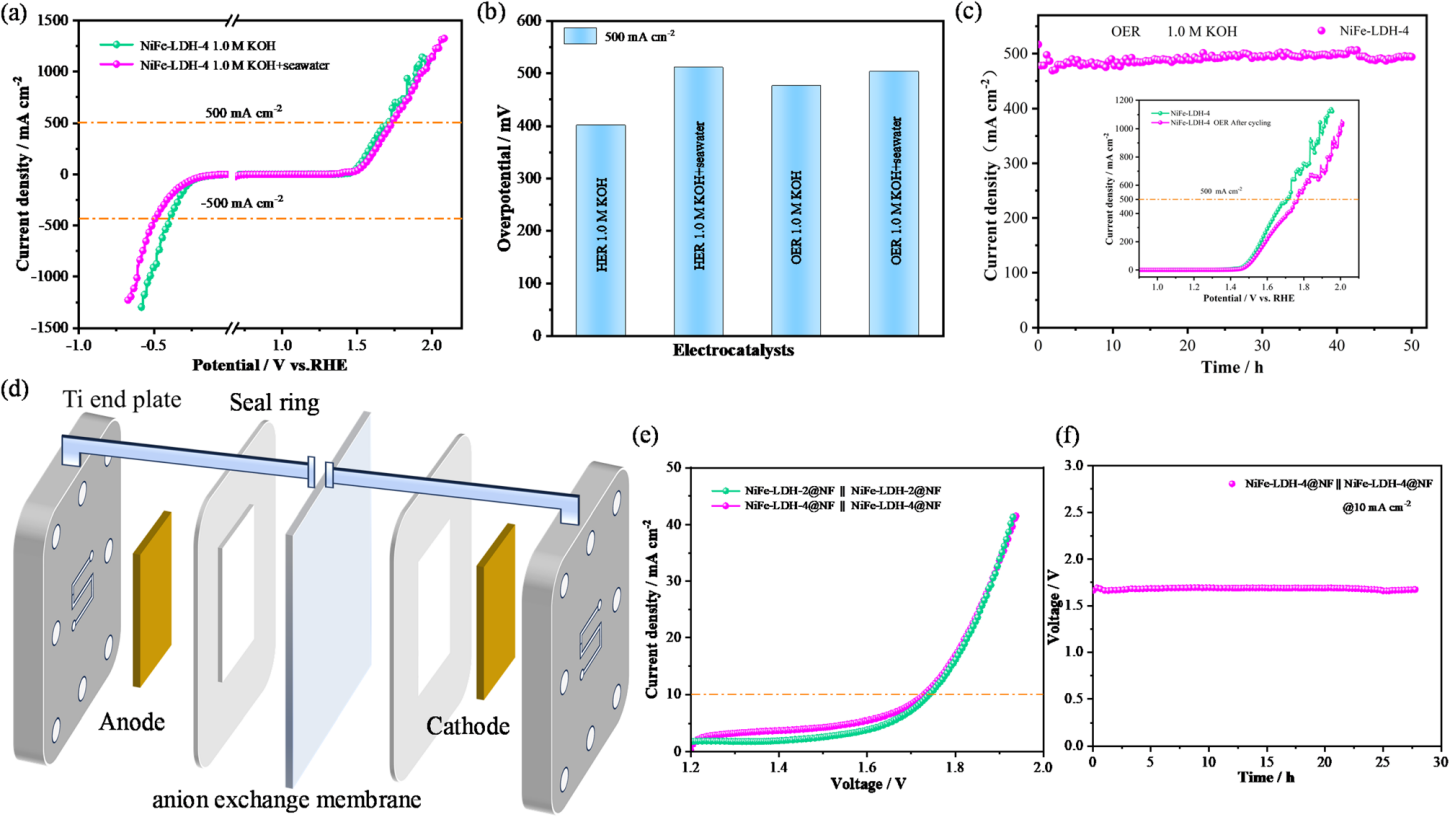
Testing of catalysts at high current density and AEMWE performance (a) LSV curves of NiFe-LDH-4 in different environments (b) overpotential bar chart of NiFe-LDH-4 under different environments (c) timing current stability curve in 1.0 M KOH, illustrated as LSV curve (d) schematic diagram of the AEM electrolyzer (e) polarization curves of electrolytic cells with different catalysts (f) NiFe-LDH-4@NF timing potential curve of AEM electrolysis cell operated by electrocatalyst.

To explore the potential industrial applications of the electrocatalyst, high current density tests were conducted under both alkaline and alkaline seawater conditions. As shown in the LSV curve (Fig. 8a) and overpotential bar chart (Fig. 8b), at a current density of 500 mA cm^−2^, the NiFe-LDH-4 exhibits an OER overpotential of 476.3 mV and a HER overpotential of 401.7 mV under alkaline conditions, while under alkaline seawater conditions, it shows an OER overpotential of 503.5 mV and a HER overpotential of 511.5 mV. Next, the durability of NiFe-LDH-4 at high current densities was evaluated, as illustrated in Fig. 8c. Under alkaline oxygen evolution conditions, the NiFe-LDH-4 catalyst demonstrates excellent stability with no significant decay in current density after 50 hours of cycling at 500 mA cm^−2^, as evidenced by the LSV curves before and after cycling shown in the inset. This indicates its potential for long-term stability even at industrial-level current densities. Fig. S4(a–c)† present the cyclic stability curves of alkaline HER, alkaline seawater OER, and HER at high current densities, respectively, showing good stability with no obvious decay in current density after 25 hours of cycling. Fig. S4d† displays the SEM images of the NiFe-LDH-4 after HER cycling in alkaline seawater, revealing no significant differences in surface morphology between the post-cycling sample and the initial sample. The good stability and lack of significant morphological changes at high current densities in alkaline seawater provide indirect evidence for the previously observed decrease in HER performance of NiFe-LDH-4 in alkaline seawater environments. This further affirms its potential application in industrial-scale seawater splitting.

Considering the good activity and stability of the NiFe-LDH-4 catalyst under alkaline conditions, the feasibility of the electrocatalyst for practical applications was further evaluated. An AEMWE (Anion Exchange Membrane Water Electrolysis) device capable of producing hydrogen in an alkaline environment was constructed as shown in Fig. 8d. The working principle is as follows: OH^−^ ions migrate from the cathode through the AEM to the anode to maintain charge balance. According to the following equation:^58^

4 Anode (OER): 4OH^−^ → O_2_↑ + 2H_2_O + 4e^−^

5 Cathode (HER): 4H_2_O + 4e^−^ → 2H_2_↑ + 4OH^−^

During testing, a 1.0 M KOH solution was used as the electrolyte. Simultaneously, pre-heated ultrapure water was pumped into the system at a flow rate of 50 mL min^−1^, and the operating temperature was maintained at 80 °C. For the polarization curve test, the upper voltage limit was set to 2.2 V. The stability of the catalyst in the practical electrolysis environment was evaluated by recording the voltage over time. The AEM electrolyzer is equipped with NiFe-LDH-4@NF (1.0 cm × 1.0 cm) as a bifunctional electrode and an anion exchange membrane. As shown in Fig. 8e, the NiFe-LDH-4@NF‖NiFe-LDH-4@NF configuration requires an electrolysis potential of 1.725 V to reach 10 mA cm^−2^, compared to a requirement of 1.742 V for the NiFe-LDH-2@NF‖NiFe-LDH-2@NF configuration. Additionally, the NiFe-LDH-4@NF‖NiFe-LDH-4@NF electrolyzer shows almost no change in electrolysis potential at 10 mA cm^−2^ over 27 hours of constant current electrolysis, as illustrated in Fig. 8f, indicating that the NiFe-LDH-4@NF‖NiFe-LDH-4@NF electrolyzer exhibits excellent stability. These results suggest that the NiFe-LDH-4@NF electrocatalyst holds significant industrial application prospects for green hydrogen production.

## 4. Conclusion

This study prepared the NiFe-LDH-4 electrocatalyst using a simple one-step hydrothermal synthesis method. Due to its unique nanosphere structure with interconnected internal nanosheets, it provides a large specific surface area, thus offering numerous electrochemically active sites for electrocatalytic reactions. The catalyst exhibited excellent OER and HER performance in both alkaline water and seawater electrolytes. In 1.0 M KOH electrolyte, it showed an oxygen evolution overpotential of only 235 mV at a current density of 50 mA cm^−2^ (175 mV@10 mA cm^−2^), along with a Tafel slope of 80.32 mV dec-1 and excellent stability. At a current density of 10 mA cm^−2^, the corresponding water splitting voltage was 1.52 V. In alkaline seawater, functioning as a bifunctional electrocatalyst, it demonstrated a hydrogen evolution overpotential of 96.5 mV and a Tafel slope of 66.97 mV dec^−1^ at −10 mA cm^−2^. Considering industrial applications, high current density tests were conducted in alkaline and alkaline seawater environments, and an AEMWE device was constructed. This research provides new insights into the rational design and construction of highly efficient electrocatalysts, laying a foundation for the development of more efficient alkaline water/seawater electrocatalytic materials.

## Conflicts of interest

The authors declare no conflict of interest.

## Supplementary Material

NA-007-D5NA00350D-s001

## Data Availability

The data that supports the findings of this study is available from the corresponding authors upon reasonable request.
