# Supplementary material for: NiFe-LDH as a bifunctional electrocatalyst for efficient water and seawater electrolysis: enhanced oxygen evolution and hydrogen evolution reactions
Source: Nanoscale Adv. 2025 Jul 30;7(18):5546–60. doi: 10.1039/d5na00350d (PMC12308914; doi:10.1039/d5na00350d)
Supplement: NA-007-D5NA00350D-s001 [file NA-007-D5NA00350D-s001.pdf]

Supplementary Information (SI) for Nanoscale Advances.
This journal is © The Royal Society of Chemistry 2025

NiFe-LDH as a Bifunctional Electrocatalyst for Efficient Water and Seawater Electrolysis: Enhanced Oxygen Evolution and Hydrogen Evolution Reactions

Xin Li[a,1], Song-lin Xu[a], Jia Li[a], Shuang-shuang Zhang[a], Bo-yao Zhang[a], Rong-da Zhao[a,*], Fu-fa Wu[a,*], De-peng Zhao[b,*]

[a]School of Materials Science and Engineering, Liaoning University of Technology, Jinzhou, Liaoning 121000, China

[b]School of New Energy, Shenyang Institute of Engineering, Shenyang, Liaoning, 110136, P. R. China

Correspondences addressed: Rongdazhaoln@126.com; hellodepeng@163.com

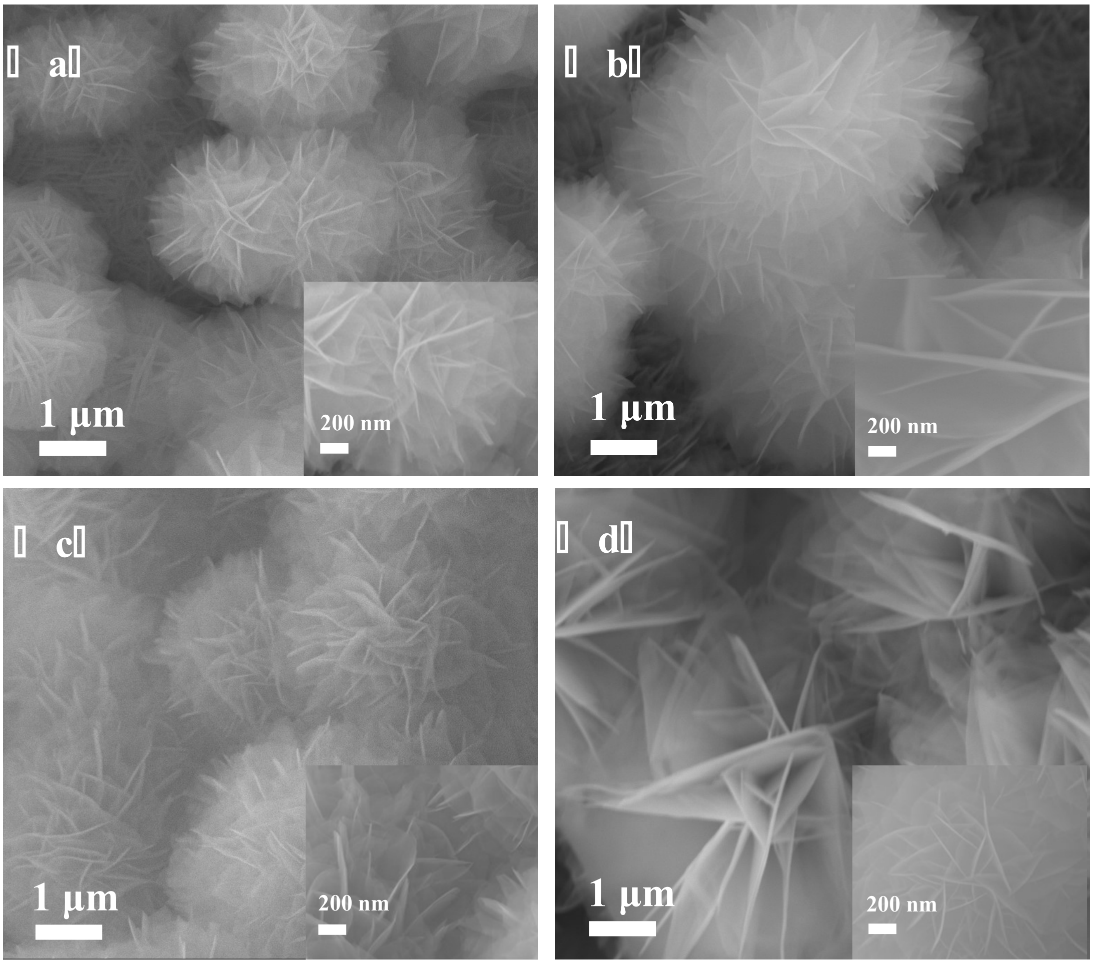

Figure S1 (a-d) **SEM images of NiFe-LDH-1, NiFe-LDH-2, NiFe-LDH-4, and NiFe-LDH-6 in sequence**

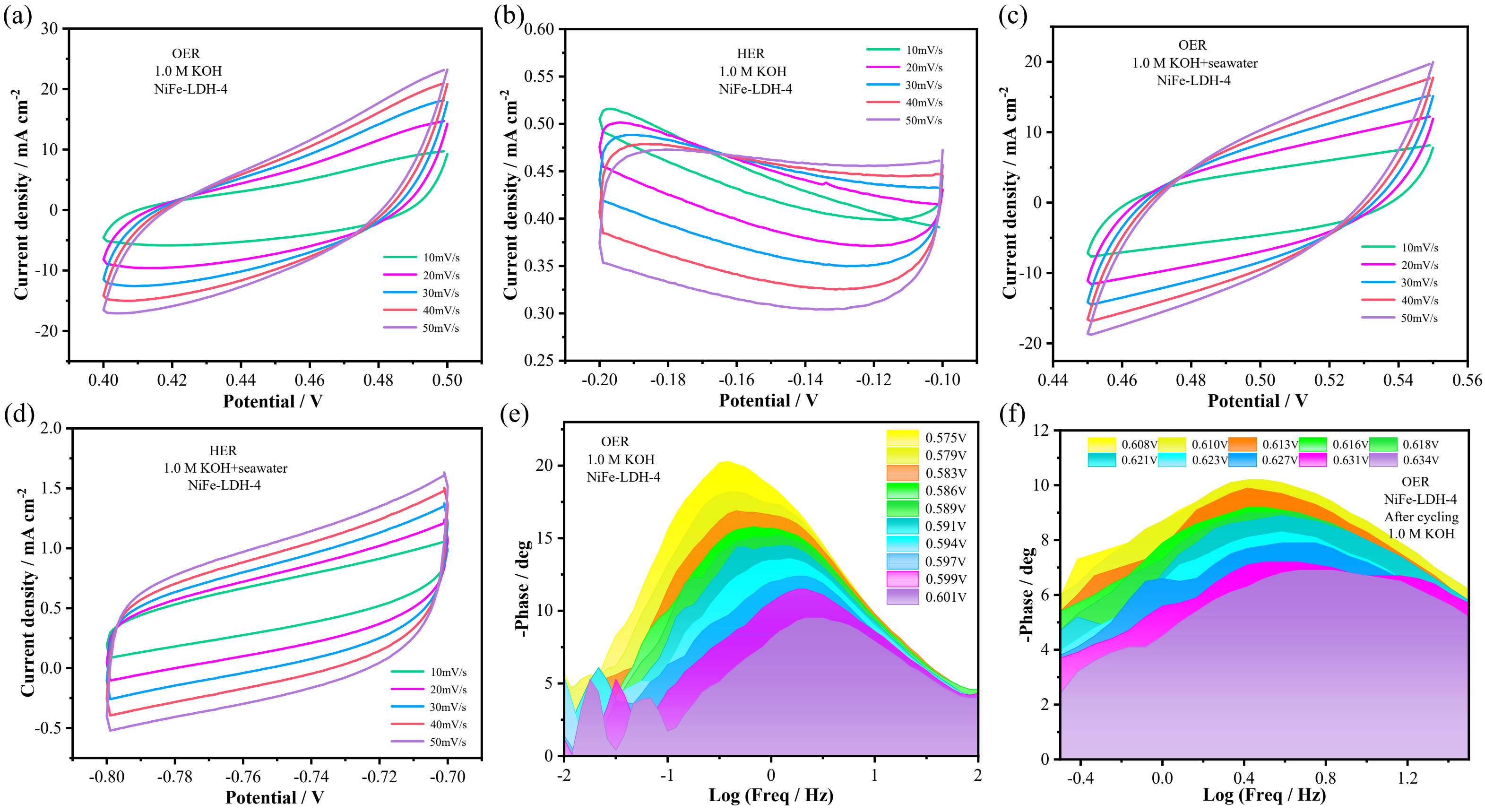

Figure S2 (a-d) CV curves. (e,f) Bode plots of NiFe-LDH-4 before and after oer cycling

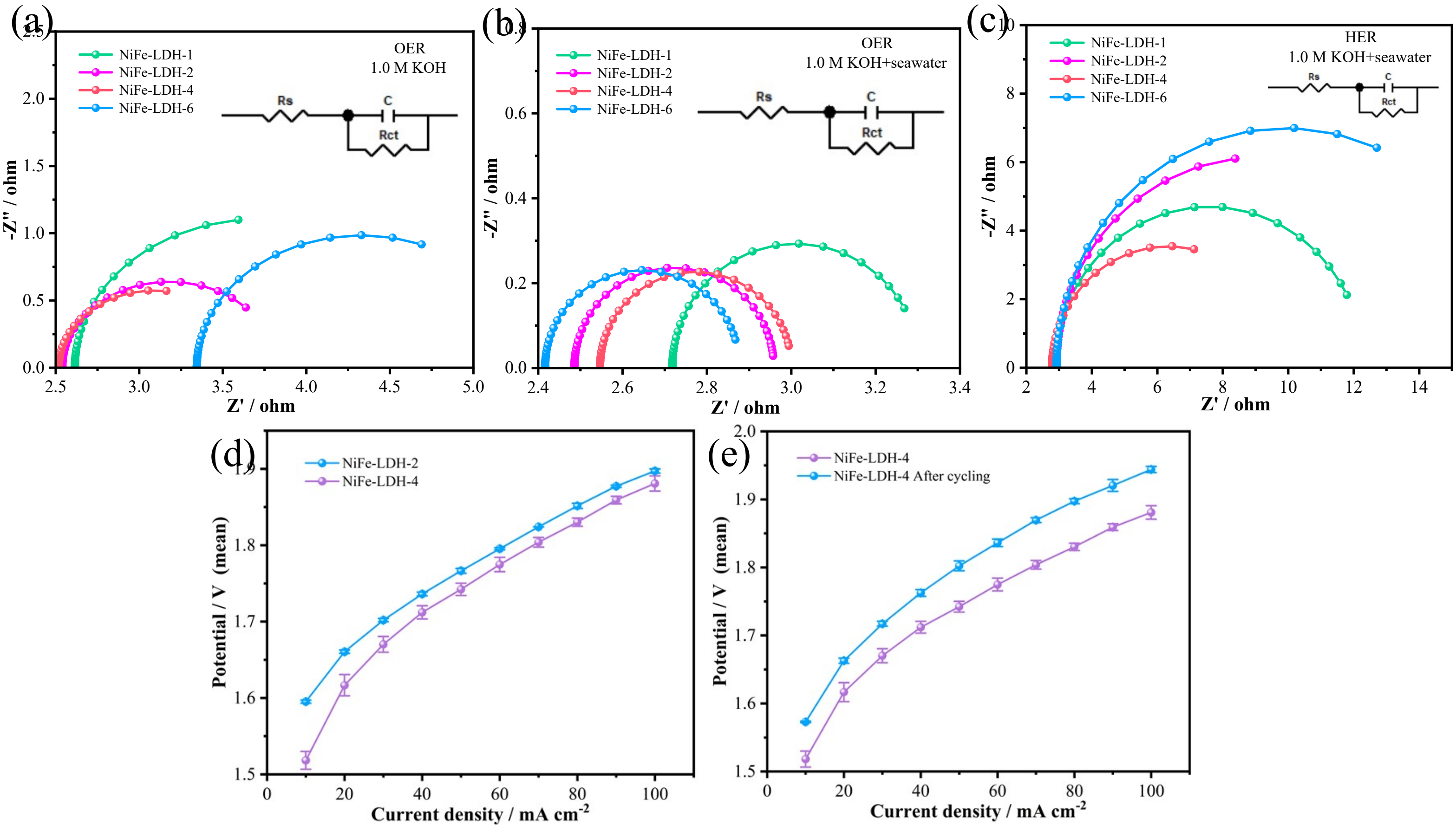

Figure S3 (a-c) Nyquist fitting curve . (e,f) Test plot of the average voltage at each current density, where the error bars represent the standard deviation from three independent measurements.

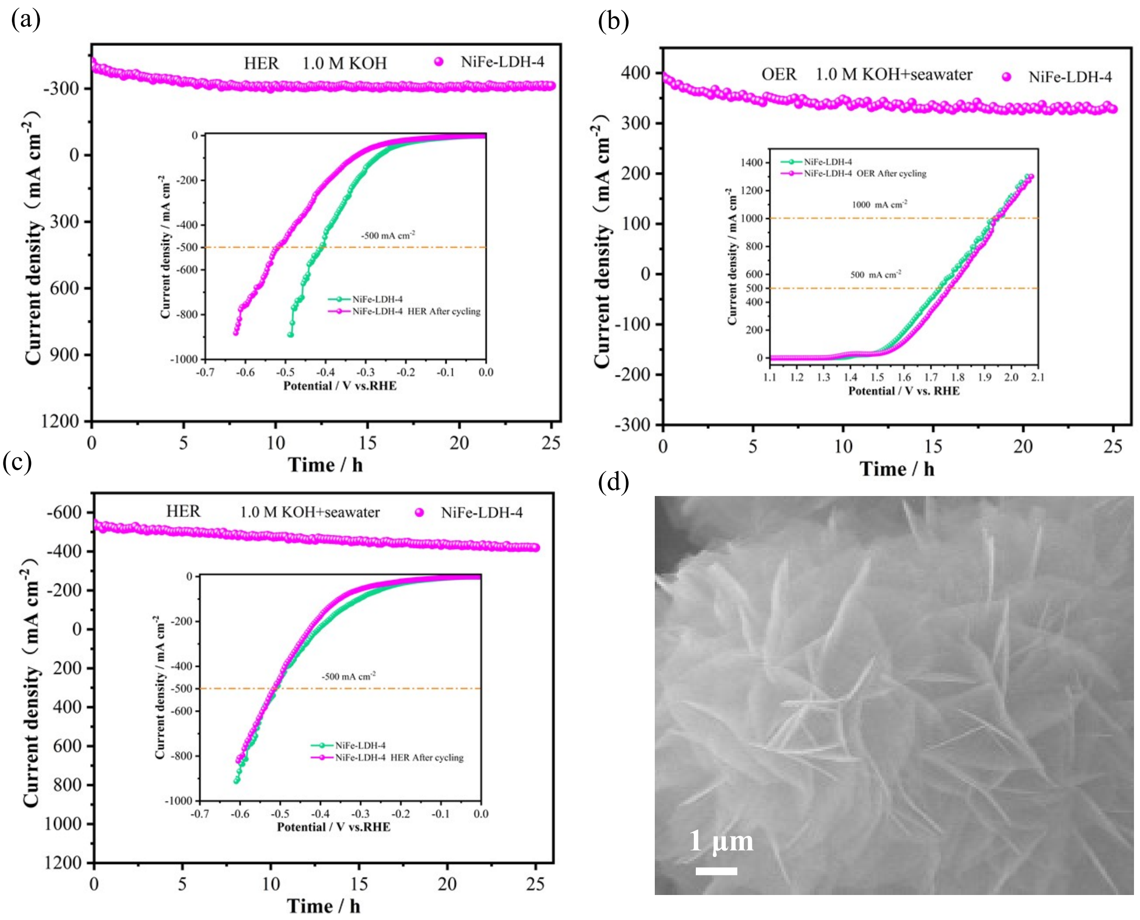

Figure S4 (a-c) Stability curves of timed currents in different environments (d) SEM image after alkaline seawater HER circulation

Tab. S1 Electrochemical impedance analysis of the as-obtained samples

Catalysts	R_s/Ω	$R_{ct}/\Omega\ cm^{-2}$
NiFe-LDH-1	2.615	2.215
NiFe-LDH-2	2.540	1.281
NiFe-LDH-4	2.518	1.150
NiFe-LDH-6	3.345	1.970

Tab. S2 Electrochemical impedance analysis of the as-obtained samples

Catalysts	R_s/Ω	$R_{ct}/\Omega\ cm^{-2}$
NiFe-LDH-1	2.718	0.586
NiFe-LDH-2	2.486	0.473
NiFe-LDH-4	2.547	0.454
NiFe-LDH-6	2.416	0.461

Tab. S3 Electrochemical impedance analysis of the as-obtained samples

Catalysts	R_s/Ω	$R_{ct}/\Omega\ cm^{-2}$
NiFe-LDH-1	2.884	9.414
NiFe-LDH-2	2.914	12.28
NiFe-LDH-4	2.784	7.093
NiFe-LDH-6	2.924	14.00
